# Attitudes Toward Deprescribing in Older Adults and Caregivers: A Survey in Quebec, Canada

**DOI:** 10.1177/07334648211069553

**Published:** 2022-03-04

**Authors:** Bianca Rakheja, Caroline Sirois, Nicole Ouellet, Barbara Roux, Marie-Laure Laroche

**Affiliations:** 1 Faculté de Médecine, 12369 Université Laval, Québec, Canada; 2 Faculté de Pharmacie, 4440 Université Laval, Québec, Canada; 3 Centre d’excellence sur le Vieillissement de Québec, Québec, Canada; 4 VITAM, Centre de Recherche en Santé durable, Québec, Canada; 5 Département des Sciences Infirmières, 14846 Université du Québec à Rimouski, Rimouski, Canada; 6 Centre de Pharmacovigilance, de Pharmacoépidémiologie et d’information sur Les Médicaments, 36715 CHU de Limoges, Limoges, France; 7 Laboratoire Vie-Santé, Faculté de Médecine de Limoges, Limoges, France

**Keywords:** deprescribing, elderly, attitudes, perceptions, polypharmacy

## Abstract

This study aimed to describe attitudes toward deprescribing among older adults and caregivers. We recruited 110 adults 65 years and above using at least one prescribed medication for at least 3 months, and 95 unrelated caregivers (18+) of older adults with such characteristics, who answered the validated French version of the revised Patients’ Attitudes Towards Deprescribing questionnaire. More older adults (84.5%) than caregivers (70.5%) (*p* = .007) would be willing to stop at least one medication if the doctor said it was possible. Conversely, 93.5% of older adults and 78.9% of caregivers were satisfied with the current medications taken (*p* = .0024). The results did not vary according to age, sex, number of medications taken, education level, or residency. Thus, older adults and caregivers are disposed to undertake deprescribing, regardless of sociodemographic characteristics. However, relying solely on satisfaction with current medications may not be sufficient to identify relevant deprescribing opportunities.

## Introduction

The aging population coupled with the rising prevalence of chronic illnesses has led to the escalation of polypharmacy, that is, the use of many medications simultaneously (Reason et al., 2012). In fact, 65.7% of Canadians over 65 years received prescriptions for at least five different drug classes in 2016 (Canadian Institute for Health Information, 2018). In addition, 26.5% of these individuals were prescribed at least 10 different drug classes with 8.4% receiving prescriptions for 15 or more different drug classes (Canadian Institute for Health Information, 2018). Polypharmacy is associated with substantial negative consequences (Davies et al., 2020; Khezrian et al., 2020; Maher et al., 2014; Wastesson et al., 2018). Non-adherence, adverse drug events, impaired functional or cognitive status, falls, urinary incontinence, declining nutritional status, and increased healthcare costs have been associated with polypharmacy (Davies et al., 2020; Khezrian et al., 2020; Maher et al., 2014; Wastesson et al., 2018). In addition, the use of potentially inappropriate medications (PIMs), that is, medications whose benefits are lower than risks, is an ongoing issue among older adults (“American Geriatrics Society 2019 Updated AGS Beers Criteria(R) for Potentially Inappropriate Medication Use in Older Adults,” 2019). Almost 50% of Canadians 65 years and older take at least one PIM, such as benzodiazepines, anticholinergic medications, or proton pump inhibitors for longer periods than requested for their medical condition (Canadian Institute for Health Information, 2018; Roux et al., 2020). Older individuals with polypharmacy are notably at greater risk of using PIMs (Roux et al., 2020). Therefore, it is evident that the issue of polypharmacy needs to be addressed.

The deprescribing of medications represents a tangible approach toward medicine cessation for healthcare providers to prevent and reduce polypharmacy thus decreasing the risk of adverse drug events (Reeve et al., 2015). Prescribers, pharmacists, nurses, and other allied healthcare professionals, are encouraged to actively undertake deprescribing interventions; they can rely on different tools to support these interventions and to provide information to patients (Reeve, 2020). However, deprescribing relies on the active involvement of older adults and their caregivers since the very essence of the process is based on shared decision-making. Indeed, understanding attitudes and beliefs is necessary to implement successful deprescribing (Pickering et al., 2020). Yet, there is still little data on the perception of deprescribing among older adults in the community, and even less for caregivers. A survey conducted in 2016 in Canada showed that only 7% of older adults were familiar with the term “deprescribing”, which is a predictor of initiating a conversation on deprescribing with a healthcare professional (Turner & Tannenbaum, 2017). With the efforts of the Canadian Deprescribing Network (www.deprescribingnetwork.ca) (Tannenbaum et al., 2017), knowledge about deprescribing has likely increased in the population, thus fostering an open-mindedness toward this process. In 2015, a study revealed that half of older adults in Canada wished to reduce the number of medications they were taking (Sirois et al., 2017), but to our knowledge, there is no recent data highlighting any such changes. In addition, the perception of caregivers has not yet been assessed in Canada. To adequately direct their efforts to optimize medication use, it is essential for health professionals to gain a better understanding of the attitudes and beliefs of older adults and caregivers toward deprescribing. Furthermore, while it is important to study and document situations of polypharmacy, it is of utmost importance to prevent polypharmacy. Hence, the goal is to use this understanding of attitudes toward deprescribing to implement it not only where polypharmacy has been established, but also where it has yet to occur.

Thus, we aimed to describe the attitudes of older adults and caregivers in Quebec, Canada, toward deprescribing, using the adapted French version of the revised Patients’ Attitudes Towards Deprescribing (rPATD) questionnaire (Reeve et al., 2016; Roux et al., 2021). As a secondary and exploratory objective, we intended to compare the attitudes toward deprescribing between older adults and caregivers to determine whether their perceptions are aligned, which would facilitate the implementation of interventions. Finally, a third exploratory objective was to assess whether certain sociodemographic characteristics are associated with (1) willingness to deprescribe and (2) satisfaction with current medications, which again would help to modulate the deprescribing interventions.

## Methods

This study was approved by the institution Ethical review boards of the *Université du Québec à Rimouski* (CER-101-745). An information sheet explaining the study and the benefits and risks of participating was provided with every questionnaire. The participants did not provide any personally identifiable information; the data collected was anonymous. Therefore, the consent was implicit when participants completed the questionnaire.

Older adults and caregivers were recruited in community pharmacies and community centers as well as through institutions for independent elders and community organizations in Quebec, Canada. A research assistant presented the study, answered any questions the individual might have, and inquired whether they would be interested to participate. The older adult interested to participate was invited to self-complete the questionnaire at this moment. In order to participate, the older adult had to be at least 65 years of age and taking one or more prescribed medication for the past 3 months. Caregivers had to be at least 18 years of age and providing care for an older adult meeting the above-stated criteria. Sample populations were recruited independently, that is, older adults and caregivers were not paired. We aimed to recruit 100 older adults and 100 caregivers.

Two versions of the French rPATD questionnaire (caregivers’ and older adults’ versions) were used, for the respective sample populations (Roux et al., 2021). In the questionnaire for older adults, the first section collected sociodemographic information to describe the participant and their current medications. The second section contained 22 statements about polypharmacy and deprescribing in addition to the participants’ involvement in the management of their medication and related decisions, and general satisfaction regarding their medications. The participants rated their agreement with each statement on a 5-point Likert scale. The version of the questionnaire for caregivers contained an additional section to characterize the caregiver, and the second section contained 19 statements to similarly measure their attitudes toward deprescribing. Once the questionnaires were completed, we entered the data into an “electronic Case Report Form” (eCRF).

### Statistical Analysis

We used descriptive statistics (proportions and means with standard deviations) to summarize the demographic characteristics and participants’ attitudes, beliefs, and experiences regarding medications, and their potential withdrawal. Exploratory analyses of the second objective were performed using chi-square tests to compare the proportions of older adults and of caregivers who agreed (strongly agree and agree) or disagreed (unsure, disagree and strongly disagree) with similar statements in the survey. For the third objective, we performed cross-tabulations using chi-square tests to explore the associations between sociodemographic characteristics (age, sex, place of residence, number of medications, education levels) and the answers to the last two general questions of the respective surveys (Questions 21–22 [older adults] and 18–19 [caregivers]), which assessed overall willingness to deprescribe and satisfaction with the current medications. All analyses were performed with significance level set at 0.05. We did not correct for multiple testing.

## Results

A total of 110 older adults and 95 caregivers responded to the survey (Table 1). The mean age of older adults was 75 years (*SD* 7.2) and they were taking a median number of 4 daily medications (IQR: 2–6). The mean age of caregivers was 69 years (*SD* 9.7) while the mean age of the care receivers was 80 years (*SD* 8.4). The care receivers were taking a median number of 5.5 different daily medications (IQR: 4–8).

**Table 1.** Characteristics of Older Adults and Caregivers and Their Care Receivers.

Characteristic	Older Adults (*n* = 110)	Caregivers (*n* = 95)	Care Receivers (*n* = 95)
**Age** (years)-mean (*SD*)^a^	75 (7.2)	69 (9.7)	80 (8.4)
**Sex—**no. (%)^b^			
Male	41 (37)	20 (21)	45 (47)
Female	69 (63)	75 (79)	49 (52)
**Relation to care receiver—**no. (%)^b^			
Spouse		48 (51)	
Child		11 (12)	
Sibling		11 (12)	
Other family member		20 (21)	
No familial relation		4 (4)	
**Highest level of education—**no. (%)^c^			
Elementary school	11 (12)	6 (6)	32 (34)
High school	26 (27)	26 (27)	14 (15)
College	32 (34)	28 (29)	30 (32)
University	39 (41)	35 (37)	16 (17)
**Residence—**no. (%)^d^			
House, apartment, or condominium	90 (82)		49 (52)
Institution	19 (17)		44 (46)
Other	1 (1)		0 (0)
**Number of daily medications—**median (IQR)^e^	4 (2–6)		5.5 (4–8)
**Medication administration aid users—**no. (%)^f^	49 (45)		51 (54)
**Medication management—**no. (%)^b^			
Self-management	100 (91)		22 (23)
Spouse or caregiver	1 (1)		19 (20)
Self-management & spouse or caregiver	2 (2)		6 (6)
Professional caregiver	7 (6)		32 (34)
Family members	0 (0)		2 (2)
Person with no familial relation	0 (0)		4 (4)
Other combinations	0 (0)		9 (9)

*Note.* SD = standard deviation, IQR = interquartile range; Percentages may not total 100 because of rounding.

^a^Data missing for one caregiver.

^b^Data missing for one care receiver.

^c^Data missing for two older adults and three care receivers.

^d^Data missing for two care receivers.

^e^Data missing for five older adults and 21 care receivers.

^f^Data missing for two older adults and 10 care receivers.

The responses of older adults and caregivers to the survey questions are presented respectively in Supplementary Tables S1 and S2 in the appendix. Briefly, only a quarter of older adults (*n* = 28) indicated a desire to try stopping one of their medications. However, most of them would be willing to stop at least one of their medications if their doctor said it was possible (*n* = 93; 85%), though 59% (*n* = 65) expressed reluctance at the idea of stopping a medication they had been taking for a long time and that the vast majority (*n* = 101; 92%) were satisfied with their current medications. Similarly, 27 caregivers (28%) conveyed that they would like the doctor to attempt stopping one of their care receiver’s medications. However, 67 (71%) agreed that they would be willing to stop at least one of the medications taken by their care receiver if the doctor said it was possible. Conversely, more than half of caregivers (*n* = 52; 55%) said they would be reluctant to stop a medication that their care receiver has been taking for a long time and four out of five (*n* = 71; 79%) were satisfied with the current medications.

**Figure 1.**
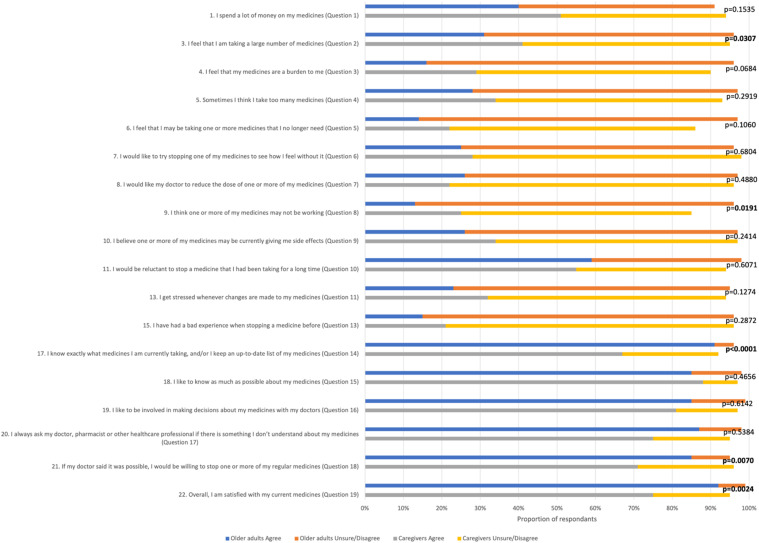
Comparison of older adults’ and caregivers’ responses to the statements of the revised Patients’ Attitudes Toward Deprescribing questionnaire. Legend: The number and the question on the left correspond to the questions posed to older adults and the question number in parentheses refers to the corresponding question in the caregiver survey. In each line, the proportion of participants who agree (strongly agree, agree) is contrasted with that of participants who are uncertain or who disagree (unsure, disagree, strongly disagree). The *p*-value results from the chi-square comparison of responses from older adults and caregivers, with a bold *p*-value indicating that the result is statistically significant (alpha = 0.05).

Figure 1 presents the answers to the corresponding questions between the group of older adults and that of caregivers. There were some statistically significant differences between the two groups based on the proportion of participants who agreed with statements in the survey. Caregivers were more likely to view medications as a burden (question 3 for older adults and question 2 for caregivers, respectively) (*p* = .0307). Likewise, the thought that “the medicine is not working” was more common among caregivers (Q9/Q8, *p* = .0191). On the other hand, older adults were more familiar with their medications versus caregivers with reference to their care receiver's medications (Q17/Q14, *p* < .0001). Older adults would be more inclined to stop a medication if the doctor told them it was possible (Q21/Q18, *p* = .0070) and were generally more satisfied with their current medication in comparison with caregivers vis-à-vis their care receiver’s current medication (Q22/Q19, *p* = .0024).

There was no statistically significant association with the studied sociodemographic charateristics and willingness to deprescribe or satisftaction with current medications (Table 2). However, male participants in the older adults group tended to report dissatisfaction with their current medications more often than females (12.5% vs. 2.9%, *p* = .0514).

**Table 2.** Responses of Older Adults and Caregivers to the Two General Questions of the Survey According to Sociodemographic Characteristics.

Characteristic	Question 21/18: If Doctor Said it was Possible, Would be Willing to Stop Medicines			Question 22/19: Overall, Satisfied with Medicines		
	Number of Participants Who Strongly Agreed/Agreed	Number of Participants Who Were Unsure/Disagreed/Strongly disagreed	*p* Value	Number of Participants Who Strongly Agreed/Agreed	Number of Participants Who Were Unsure/Disagreed/Strongly disagreed	*p* Value
**Older Adults**						
**Age** (year)**—**no.						
65–69	31	3	0.7985	33	2	0.7572
70–74	20	2		20	2	
75–79	20	4		24	1	
80–84	9	2		11	2	
85+	12	1		13	0^a^	
**Sex—**no.						
Male	36	3	0.3545	35	5	0.0514
Female	57	9		66	2	
**Number of medications taken—**no.						
1	10	2	0.2948	22	0^a^	0.2249
2–4	44	3		44	3	
5–9	29	5		34	2	
10+	5	2		6	2	
**Education—**no.						
Primary	7	3	0.2881	9	2	0.3473
High school	25	4		29	1	
College	22	3		25	1	
University	37	3		36	3	
**Residence**–no.						
House	79	8	0.0907	82	6	0.8037
Institution**—**	13	4		18	1	
**CAREGIVERS**						
**Age of caregiver** (year)**—**no.			0.3140			0.2129
≤60	12	0^a^		7	5	
61–70	25	12		20	8	
71–80	25	11		31	5	
81+	5	1		4	1	
**Sex of caregiver—**no.						
Male	12	5	0.7526	14	3	0.6600
Female	55	19		55	16	
**Relation to care receiver—**no.			0.4724			0.2613
Spouse	33	12		36	8	
Child	9	1		8	2	
Sibling	6	5		9	2	
Other family	14	6		16	2	
Not family	4	0^a^		1	2	
**Education of caregiver—**no.						
Elementary	5	0^a^	0.5169	3	1	0.2465
High school	17	10		24	2	
College	20	5		19	6	
University	25	10		25	10	
**Age of care receiver—**no.						
65–69	8	4	0.3619	11	1	0.3889
70–74	6	5		8	3	
75–79	14	3		11	6	
80–84	20	4		18	5	
85+	19	8		23	4	
**Sex of care receiver—**no.			0.7735			0.9679
Male	33	11		34	9	
Female	34	13		37	10	
**Number of medications taken by care receiver—**no.						
1	4	0^a^	0.0639	4	0^a^	0.8434
2–4	9	9		14	3	
5–9	28	7		29	6	
10+	13	1		10	4	
Unknown	7	3		7	3	
**Education of care receiver—**no.						
Primary	24	8	0.7722	25	7	0.9996
High school	20	10		23	6	
College	11	3		11	3	
University	11	3		11	3	
**Residence of care receiver—**no.						
House	35	12	0.7991	36	11	0.4295
Institution	31	12		35	7	

^a^When values of zero were present, the value 1 was entered in the calculation of chi-square, so that it could be performed.

## Discussion

The results show that older adults and caregivers appear to be open to deprescribing, but their beliefs and degrees of readiness are incongruous. Caregivers’ attitudes toward deprescribing are not as inclined, perhaps due to their strong beliefs that deprescribing may be neglectful. Overall, none of the examined sociodemographic factors were associated with specific perceptions on deprescribing.

Our results concur with the conclusion of a recent systematic review of 29 studies that states that most older adults and caregivers are willing to deprescribe medications (Chock et al., 2021). According to this review, if deemed possible by their doctor, 87.6% (95% CI: 83.3–91.4) of older adults would be willing to stop one medication (Chock et al., 2021). Willingness to deprescribe was lower among caregivers (74.8%; 49.8–93.8) (Chock et al., 2021). However, some specific studies showed higher proportion of willingness to deprescribe among caregivers (Kua et al., 2021; Reeve et al., 2019), such as an Australian study where this proportion reached 84% (Reeve et al., 2019). Interestingly, the overall open-mindedness of older adults toward deprescribing in our study is superior to what was seen in a survey taken in 2016 in Canadians, which showed that 72.4% of community-dwelling older adults were willing to stop one or more of their medications if their doctor said it was possible (Sirois et al., 2017). That said, it would be interesting to observe caregivers’ attitudes in the Canadian community over time to see if they follow the same trend given that deprescribing is becoming more common.

The differences in willingness to deprescribe and satisfaction with the current medication were remarkable between the two groups. These findings need to be explored with paired analysis to determine if the differences are contextual, or, conversely, if they truly represent differences in perspective. This is important for deprescribing, because social support is a key component to the success of the intervention. In our survey, the majority of caregivers were older adults themselves, indicating that they may also be facing the issues of polypharmacy. This theory may explain the similarities in terms of general open-mindedness toward deprescribing seen in both groups. However, their views are not completely compatible perhaps because perceptions tend to differ when a situation concerns someone else versus oneself. The contrast between older participants’ and caregivers’ perspectives toward deprescribing may also arise from certain differing characteristics between the surveyed older adults and the care receivers which were not assessed in our questionnaires. However, the observed differences in residential context and medication management when comparing the surveyed older adults and the care receivers suggests that the latter are overall less autonomous.

Although they expressed a high level of satisfaction with the current medications, a large proportion of older adults and caregivers would be willing to stop at least one medication if the doctor deemed it possible. This finding appears to illustrate the importance of the professional–patient relationship and shared decision making in the implementation of deprescribing interventions. Incidentally, both caregivers and older adults have a substantial interest in being involved in decision-making regarding medications. This conjecture coincides with a recent qualitative study which showed that both subgroups, though caregivers in particular, place an importance on the relationship with the prescriber (Pickering et al., 2020). In addition, caregivers and older adults had different viewpoints on certain aspects regarding “medication value,” and health professionals should consider the beliefs of both subgroups when instigating a discussion for successful deprescribing (Pickering et al., 2020). Given that deprescribing is a multidisciplinary process, it involves many professionals such as the prescriber, the pharmacist, the nurse and the social worker alongisde the patient and their caregivers. These professionals play fundamental roles in exploring patient and caregiver attitudes toward medications and possible deprescribing of said medications, in addition to providing education and support. Health professionals would therefore be better equipped to intervene at all stages of the deprescribing process if they were aware of patients’ and caregivers’ perceptions and attitudes, allowing them to form a genuine alliance in accordance with the person-centered approach.

Other statistically significant differences between older adults and caregivers, based on the proportion of participants who agreed with statements of the survey, may be explicable. Responses to certain statements, such as #3 and #9 in the older adult survey (#2 and #8 in the caregiver survey) may depend on the context. However, since the participants were not paired, they may not represent true differences. In addition, it is understandable that a caregiver, as opposed to an older adult managing their own medication, may not have as detailed of an understanding of their care receiver's medication (question 17/14).

Conversely, the issues of polypharmacy and medication burden did not differ between groups, and they were not highlighted by the participants’ responses. Contrastingly, a systematic review published in 2016 noted that medication burden, among the various burdens experienced by patients, was one of the most frequently mentioned (Mohammed et al., 2016). However, the study recognized that some patients felt overwhelmed and as though they were losing control due to the number of medications they were taking, yet others perceived their medications as essential for their health without regard to the number itself (Mohammed et al., 2016). The latter point of view may explain the survey results with respect to this concept. Also, our population was not exclusively made up of individuals with polypharmacy; certain participants may have experienced lesser medication burden.

No differences were observed according to sociodemographic characteristics, suggesting that deprescribing could be relevant for all older adults. The factors associated with willingness to deprescribe are inconsistent between studies. In Japan, for example, increasing age and polypharmacy were associated with the willingness to deprescribe (Aoki et al., 2019). However, there was no association with sex, age, and number of medications among multimorbid older adults with polypharmacy in a study conducted in Switzerland (Rozsnyai et al., 2020). The number of medications taken has also had inconsistent associations with willingness to deprescribe (Kua et al., 2019; Reeve et al., 2013, 2018, 2019). Higher level of education has been associated with willingness to deprescribe in other countries (Aoki et al., 2019; Komagamine et al., 2018; Reeve et al., 2018), but this was not the case in our study. The setting of the study and the basic characteristics of the population studied could possibly explain some of the discrepancies found between the studies, especially when some factors, such as the level of frailty and autonomy, are not well studied. Overall, since there is no consistency between studies, clinicians can at this time assume that deprescribing may be applicable to all older adults.

There are a few limitations in the present study. The survey collected only quantitative data and could not take into account any qualitative comments. In addition, the number of medications was self-reported, which led to missing data or invalid responses. Many participants responded with a range of values, which was not accepted by the eCRF. Likewise, some questions and statements could be ambiguous for many individuals leading to different interpretations. For instance, in the following statement, *“I would like to try stopping one of my medicines to see how I feel without it*,” it may be unclear whether the doctor or the patient is stopping the medication. Also, a volunteer bias may have occurred as individuals who were more informed as well as those who were dissatisfied with their medication may have been more inclined to participate in the study. Our statistical analyses should also be considered exploratory: we conducted several analyses on a small sample and did not correct for multiple tests. In addition, since univariate analyses were not found to be statistically significant, no multivariate analysis was performed. Availability of social support, a variable that may be significant for deprescribing, was not included in the survey. Likewise, ethnicity and gender (as a concept distinct from sex) were not included, but the results were unlikely modified due to this omission given that the older population in Quebec is mostly homogeneous for those variables. Finally, a more complex survey, with follow-up questions using branching logic, could have elucidated the rationale behind the respondents’ answers.

Care receivers were not surveyed in this study. Thus, paired comparison analyses to understand the precise differences between caregivers and their care receivers in a unique context of care were not possible. However, surveying two independent groups did allow certain advantages. We were able to recruit a larger number of participants, including caregivers who care for older adults with cognitive disorders, and ensure that the responses received were completely independent, which would have been logistically complex in a caregiver–care receiver study. Subsequent studies assessing and comparing caregiver and care receiver perceptions would be interesting. Likewise, it would be interesting to explore if the presence of a cognitive disorder in the care receiver affects the attitudes of the caregiver. In all cases, any disparity between the attitudes of older adults and of caregivers may render the implementation of deprescribing more complex and bias compliance to the deprescription.

It seems that both older adults and caregivers are increasingly disposed to undertake deprescribing in Quebec, though their perceptions differ on certain aspects. However, it is apparent that both subgroups maintain some reluctance toward the cessation of medications, indicating that certain barriers need to be addressed. There is no noticeable difference between attitudes toward deprescribing according to the studied characteristics of the older adults and the caregivers. There is therefore an opportunity for health professionals to integrate deprescribing extensively into their clinical practice. The fact that there is no systemic resistance toward deprescribing should be explicitly conveyed when training healthcare professionals. Additionally, there are various levers for transformation, including trust in the prescriber. A true change in the culture of medication use can be implemented by involving the patient, their caregivers and the healthcare team. Further studies should be conducted to probe clinicians' perceptions of these findings, and to determine how shared decision-making with the broader medical team, the patient, and their caregivers fits into their clinical practice.

## Supplemental Material

sj-pdf-1-jag-10.1177_07334648211069553 – Supplemental Material for Attitudes Toward Deprescribing in Older Adults and Caregivers: A Survey in Quebec, Canada Click here for additional data file. Supplemental Material, sj-pdf-1-jag-10.1177_07334648211069553 for Attitudes Toward Deprescribing in Older Adults and Caregivers: A Survey in Quebec, Canada by Bianca Rakheja, Caroline Sirois, Nicole Ouellet, Barbara Roux and Marie-Laure Laroche in Journal of Applied Gerontology
