# Supplementary material for: Attitudes Toward Deprescribing in Older Adults and Caregivers: A Survey in Quebec, Canada
Source: J Appl Gerontol. 2022 Mar 4;41(5):1376–84. doi: 10.1177/07334648211069553 (PMC9024025; doi:10.1177/07334648211069553)
Supplement: sj-pdf-1-jag-10.1177_07334648211069553 – Supplemental Material for Attitudes Toward Deprescribing in Older Adults and Caregivers: A Survey in Quebec, Canada [file sj-pdf-1-jag-10.1177_07334648211069553.pdf]

APPENDIX

Supplementary Table S1: Responses to statements from the survey for older adults (n=110)

	Number of participants who Strongly Agreed/ Agreed N (%)	Number of participants who had No opinion/ Were Unsure N (%)	Number of participants who Strongly Disagreed/ Disagreed N (%)	Number of participants with No response/ Multiple responses N (%)
1. I spend a lot of money on my medicines	44 (40%)	7 (6%)	50 (45%)	9 (8%)
2. Taking my medicines every day is very inconvenient	22 (20%)	5 (5%)	77 (70%)	6 (5%)
3. I feel that I am taking a large number of medicines	34 (31%)	6 (5%)	66 (60%)	4 (4%)
4. I feel that my medicines are a burden to me	18 (16%)	6 (5%)	82 (75%)	4 (4%)
5. Sometimes I think I take too many medicines	31 (28%)	5 (5%)	70 (64%)	4 (4%)
6. I feel that I may be taking one or more medicines that I no longer need	15 (14%)	11 (10%)	80 (73%)	4 (4%)
7. I would like to try stopping one of my medicines to see how I feel without it	28 (25%)	10 (9%)	68 (62%)	4 (4%)
8. I would like my doctor to reduce the dose of one or more of my medicines	29 (26%)	10 (9%)	68 (62%)	3 (3%)
9. I think one or more of my medicines may not be working	14 (13%)	15 (14%)	76 (69%)	5 (5%)
10. I believe one or more of my medicines may be currently giving me side effects	29 (26%)	2 (2%)	76 (69%)	3 (3%)
11. I would be reluctant to stop a medicine that I had been taking for a long time	65 (59%)	4 (4%)	38 (35%)	3 (3%)
12. If one of my medicines	48 (44%)	7 (6%)	49 (45%)	6 (6%)

	Number of participants who Strongly Agreed/ Agreed N (%)	Number of participants who had No opinion/ Were Unsure N (%)	Number of participants who Strongly Disagreed/ Disagreed N (%)	Number of participants with No response/ Multiple responses N (%)
was stopped, I would be worried about missing out on future benefits				
13. I get stressed whenever changes are made to my medicines	25 (23%)	8 (7%)	72 (65%)	5 (5%)
14. If my doctor recommended stopping a medicine, I would feel that he/she was giving up on me	15 (14%)	3 (3%)	87 (79%)	5 (5%)
15. I have had a bad experience when stopping a medicine before	17 (15%)	4 (4%)	85 (77%)	4 (4%)
16. I have a good understanding of the reasons I was prescribed each of my medicines	99 (90%)	2 (2%)	7 (6%)	2 (2%)
17. I know exactly what medicines I am currently taking, and/or I keep an up-to-date list of my medicines	100 (91%)	1 (1%)	4 (4%)	5 (5%)
18. I like to know as much as possible about my medicines	94 (85%)	1 (1%)	13 (12%)	2 (2%)
19. I like to be involved in making decisions about my medicines with my doctors	94 (85%)	3 (3%)	12 (11%)	1 (1%)
20. I always ask my doctor, pharmacist or other healthcare professional if there is something I don’t understand about my medicines	96 (87%)	2 (2%)	10 (9%)	2 (2%)
21. If my doctor said it was possible, I would be willing to stop one or more of my	93 (85%)	6 (5%)	6 (5%)	5 (5%)

	Number of participants who Strongly Agreed/ Agreed N (%)	Number of participants who had No opinion/ Were Unsure N (%)	Number of participants who Strongly Disagreed/ Disagreed N (%)	Number of participants with No response/ Multiple responses N (%)
regular medicines				
22. Overall, I am satisfied with my current medicines	101 (92%)	5 (5%)	2 (2%)	2 (2%)

Supplementary Table S2: Responses to statements from the survey for caregivers (n=95)

	Number of participants who Strongly Agreed/ Agreed N (%)	Number of participants who had No opinion/ Were Unsure N (%)	Number of participants who Strongly Disagreed/ Disagreed N (%)	Number of participants with No response/ Multiple responses N (%)
1. My care receiver's medicines are quite expensive	48 (51%)	15 (16%)	26 (27%)	6 (6%)
2. I feel that the person I care for is taking a large number of medicines	39 (41%)	10 (11%)	43 (45%)	3 (3%)
3. I feel that my care receiver's medicines are a burden to them	28 (29%)	6 (6%)	53 (56%)	8 (8%)
4. Sometimes I think the person I care for takes too many medicines	32 (34%)	3 (3%)	53 (56%)	7 (7%)
5. I feel that the person that I care for may be taking one or more medicines that they no longer need	21 (22%)	18 (19%)	52 (55%)	4 (4%)
6. I would like the doctor to try stopping one of my care receiver’s medicines to see how they feel without it	27 (28%)	14 (15%)	52 (55%)	2 (2%)
7. I would like the doctor to reduce the dose of one or more of my care receiver’s medicines	21 (22%)	14 (15%)	57 (60%)	3 (3%)
8. I think one or more of my care receiver’s medicines may not be working	24 (25%)	18 (19%)	48 (51%)	5 (5%)
9. I think one or more of my care receiver’s medicines may be currently giving them side effects	32 (34%)	18 (19%)	42 (44%)	3 (3%)
10. I would be reluctant to stop one of my care receiver’s medicines that they had been taking for a long time	52 (55%)	14 (15%)	25 (26%)	4 (4%)
11. I get stressed whenever	30 (32%)	7 (7%)	52 (55%)	6 (6%)

	Number of participants who Strongly Agreed/ Agreed N (%)	Number of participants who had No opinion/ Were Unsure N (%)	Number of participants who Strongly Disagreed/ Disagreed N (%)	Number of participants with No response/ Multiple responses N (%)
changes are made to my care receiver's medicines				
12. I feel that if I agreed to stopping one of my care receiver's medicines, then this is giving up on them	31 (33%)	5 (5%)	56 (59%)	3 (3%)
13. The person that I care for has had a bad experience when stopping a medicine before	20 (21%)	10 (11%)	61 (64%)	4 (4%)
14. I know exactly what medicines the person that I care for is currently taking and/or I have an up-to-date list of their medicines	64 (67%)	2 (2%)	23 (24%)	6 (6%)
15. I like to know as much as possible about my care receiver's medicines	84 (88%)	0 (0%)	9 (9%)	2 (2%)
16. I like to be involved in making decisions about my care receivers medicines with their doctors	77 (81%)	4 (4%)	11 (12%)	3 (3%)
17. I always ask the doctor, pharmacist or other healthcare professional if there is something I don't understand about my care receiver's medicines	71 (75%)	5 (5%)	14 (15%)	5 (5%)
18. If their doctor said it was possible, I would be willing to stop one or more of my care receiver's medicines	67 (71%)	6 (6%)	18 (19%)	4 (4%)
19. Overall, I am satisfied with my care receiver's current medicines	71 (75%)	9 (9%)	10 (11%)	5 (5%)
